# Level I and II deficits—A clinical survey on international practice of awake craniotomy and definitions of postoperative “major” and “minor” deficits

**DOI:** 10.1093/noajnl/vdae206

**Published:** 2024-11-30

**Authors:** Manuela Vooijs, Faith C Robertson, Sarah E Blitz, Christine Jungk, Sandro M Krieg, Philippe Schucht, Steven De Vleeschouwer, Arnaud J P E Vincent, Mitchel S Berger, Brian V Nahed, Marike L D Broekman, Jasper K W Gerritsen

**Affiliations:** Department of Neurosurgery, Massachusetts General Hospital, Boston, Massachusetts, USA; Department of Neurosurgery, Massachusetts General Hospital, Boston, Massachusetts, USA; Department of Neurosurgery, Massachusetts General Hospital, Boston, Massachusetts, USA; Department of Neurosurgery, Medical Faculty, Heidelberg University, Heidelberg, Germany; Department of Neurosurgery, Medical Faculty, Heidelberg University, Heidelberg, Germany; Department of Neurosurgery, Bern University Hospital, Bern, Switzerland; Department of Neurosurgery, UZ Leuven, Belgium; Department of Neurosurgery, Erasmus Medical Center, Rotterdam, The Netherlands; Department of Neurological Surgery, University of California, San Francisco, California, USA; Department of Neurosurgery, Massachusetts General Hospital, Boston, Massachusetts, USA; Department of Neurosurgery, Haaglanden Medical Center, The Hague, The Netherlands; Department of Neurological Surgery, University of California, San Francisco, California, USA; Department of Neurosurgery, Erasmus Medical Center, Rotterdam, The Netherlands

**Keywords:** awake craniotomy, complications, glioma, neurological morbidity, survey

## Abstract

**Background:**

Awake craniotomy (AC) is a technique that balances maximum resection and minimal postoperative deficits in patients with intracranial tumors. To aid in the comparability of functional outcomes after awake surgery, this study investigated its international practice and aimed to define categories of postoperative deficits.

**Methods:**

A survey was distributed via neurosurgical networks in Europe (European Association of Neurosurgical Societies, EANS), the Netherlands (Nederlandse Vereniging voor Neurochirurgie, NVVN), Belgium (Belgian Society of Neurosurgery, BSN), and the United States (Congress of Neurological Surgeons, CNS) between April 2022 and April 2023. Questions involved decision-making, including patient selection, anxiety assessment, and termination of resection. Interpretation of “major” and “minor” deficits, respectively labeled “level I” and “level II,” was assessed.

**Results:**

Three hundred and ninety-five neurosurgeons from 46 countries completed the survey. Significant heterogeneity was found in the domains of indications, anxiety assessment, seizure management, and termination of resection. Moreover, the interpretation of “major” deficits mainly included language and motor impairments. Analysis across deficit categories showed significant overlap in the domains of executive function, social cognition, and vision. Secondly, “minor” deficits and “minor cognitive” deficits showed vast overlap.

**Conclusions:**

This survey demonstrates high variability between neurosurgeons in AC practice across multiple domains, inviting international efforts to reach a consensus regarding the standardization and grading of postoperative deficits. The proposed categories of “level I” and “level II” deficits may aid in this standardization. It allows for systematic assessment of the benefit of surgery in neuro-oncology patients and allows for comparison of surgical outcomes between institutions and surgeons. This may help to optimize international guidelines for surgical neuro-oncology, including AC.

## Lay Summary

Some brain surgeons operate on patients while they are awake. This allows them to communicate with the patients during the surgery to protect important brain functions like movement and speech. However, not all surgeons perform this procedure in the same way. The authors of this study wanted to see how different surgeons approach awake surgeries. They surveyed 395 brain surgeons from 46 countries to learn about their methods and the challenges patients might face afterward. The results showed that surgeons differ in how they select patients, assess their anxiety, and decide when to stop the surgery. Most surgeons identified issues with language and movement as major challenges for patients after surgery, but there was less agreement on what should be considered a minor challenge.

**Key Points**

- This clinical survey found substantial variability between neurosurgeons regarding perioperative decision-making in awake craniotomy, including patient selection, anxiety assessment, postoperative QoL assessment, and interpretation of transient deficits.
- Neurosurgeons differed in their opinion on the interpretation of postoperative “minor” and “major” deficits.
- A graded and standardized deficit system may aid assessing the benefit of surgery in neuro-oncology patients and allows for systematic comparison of surgical outcomes 

**Importance of the Study**

Awake craniotomy with intraoperative brain mapping is a well-established method for resection of intracranial lesions. It aims to balance safe resection while preserving neurological function. Postoperatively, neurological function can be objectified with the National Institutes of Health Stroke Scale and the Neurologic Assessment in Neuro-Oncology. With the recent introduction of supramaximal resection, it becomes increasingly important to define and to standardize neurological deficits. A “graded” and standardized neurological deficit system may aid in assessing the benefit of surgery in neuro-oncology patients. Furthermore, it can be helpful when comparing surgical outcomes across neuro-oncological studies. For these reasons, this study has aimed to assess the interpretation of categorized deficits—“major” or “level I” deficits, “minor” or “level II” deficits, and “minor cognitive” deficits—of neurosurgeons. Moreover, this study evaluated the international practice heterogeneity regarding awake craniotomy and identified areas that could potentially benefit from international consensus. 

Awake craniotomy (AC) is a technique that aims to balance maximal resection with the preservation of neurological function.^1^ Today, intraoperative mapping in combination with having the patient awake during resection is a well-established method to safely maximize the extent of resection (EOR) for intraaxial tumors in or near functional brain tissue.^1^ Several studies have demonstrated that AC is a safe and feasible technique for patients with gliomas and is associated with improved EOR, improved Karnofsky Performance Status (KPS) score, decreased postoperative neurological complications, and improved survival outcomes in comparison to craniotomy under general anesthesia.^2–11^ Currently, outcome measurement scales such as the National Institutes of Health Stroke Scale (NIHSS) and the Neurologic Assessment in Neuro-Oncology (NANO) are used to objectively assess postoperative neurologic function in neuro-oncology patients.^12,13^ However, these scales do not differentiate between “minor” and “major neurological deficits, even though this distinction could have profound implications for the surgical decision-making in these patients. Pushing the boundaries of the resection in a safe manner has become even more relevant with the recent introduction of “supramaximal resection” (SMR).^14,15^ AC may be an effective surgical strategy to supplement SMR, especially in patients with tumors in or near functional tissue areas to preserve important functions while removing the additional non-contrast-enhancing part of the tumor.^10,11,16,17^ Decision-making regarding SMR includes the preoperative neurological status of a patient and the expected status postoperatively, alongside factors such as oncological markers. In gliomas, for example, overall survival after resection varies according to different isocitrate dehydrogenase-1 (IDH1) phenotypes.^18,19^ A system of graded postoperative deficits would enable 1) systematic assessment of the benefit of surgery in neuro-oncology patients, and 2) objective comparison of surgical outcomes between treatment arms, cohorts, and centers. Therefore, this study proposes a first categorization of neurological deficits: “major” (or “level I” deficits), and “minor” (or “level II” deficits), and “minor cognitive” deficits; and explores their interpretation using an international clinical survey. Additionally, the perioperative decision-making regarding awake surgery is assessed with questions on indications, patient selection, reasons for discontinuation of the awake surgery, intraoperative decision-making, and quality of life (QoL). This study may serve as a stepping stone toward international consensus on minor and major postoperative deficits.

## Methods

### Data Collection

An electronic survey was created and disseminated using REDCap (Research Electronic Data Capture) hosted at Massachusetts General Incorporated.^20,21^ The survey was distributed per email to neurosurgeons worldwide via the American Association of Neurological Surgeons (AANS), the European Association of Neurosurgical Societies (EANS), the Congress of Neurological Surgeons (CNS), the European and North American Consortium and Registry for Intraoperative Stimulation Mapping (ENCRAM), the Dutch Neurosurgical Association (NVVN), and the Belgian Society of Neurosurgery (BSN). In the electronic distribution of the questionnaire, neurosurgeons were asked to forward the survey to their personal neurosurgical network. The survey was distributed from April 2022 to April 2023 and monthly reminders were sent during this period. Participation in the survey was voluntary, without reward or reimbursement, and no personally identifiable data was collected.

The survey consisted of 25 questions regarding indications, factors in preoperative and intraoperative decision-making, stopping points, QoL, and definitions of deficits. Deficits were categorized as “major” or “level I,” “minor” or “level II.” An extra category was created—“minor cognitive” deficit—to identify whether the addition of “cognitive” would alter the interpretation of this category of deficits. Information about the neurosurgeon, including experience and number of awake craniotomies performed per year, was also obtained. The complete questionnaire can be found in Data Supplementary 1.

### Statistical Analysis

Survey data were exported for data analysis on May 6, 2023, from REDCap, and further analyzed using *R* version 4.1.1 (the *R* foundation). Continuous variables are presented as means ± standard deviations (SDs) for parametric data and medians with interquartile ranges (IQRs) for non-parametric data. Categorical variables are presented as percentages (%) and frequencies (in n). Perioperative factors in decision-making regarding AC were assessed using a Likert scale, with 5 indicating “highest importance” and 1 indicating “lowest importance.” Data was dichotomized to compare subgroups based on baseline characteristics, including World Health Organization (WHO) region, institution, neurosurgical training, and number of awake craniotomies performed. Response differences were analyzed using the χ 2 test for proportions. The Fisher’s exact test was used in case of <5 responses. Categorical questions with multiple answers were dichotomized and subsequently analyzed using similar statistical tests. Finally, definitions of “major,” “minor,” and “minor cognitive deficits” were analyzed using the χ 2 test for homogeneity. *P*-values below .05 were considered statistically significant and the Bonferroni method was applied to correct for multiple testing.

## Results

The survey yielded a total of 395 responses from 46 countries. The demographics of the respondents are summarized in Table 1 (Data Supplementary 2). 24.4% of the responses originated from Europe (*n* = 93) and 50.1% from the United States and Canada (*n* = 191). Most of the respondents were employed at an academic practice or university hospital (64.8%, *n* = 247) and had more than 5 years of experience (87.9%, *n* = 335). 23 respondents had never performed an AC (6%), while 3 respondents had performed over 100 awake surgeries (0.8%). The remaining respondents had performed 1–5 (44.1%, *n* = 168), 6–20 (34.4%, *n* = 131), and 21–50 (12.1%, *n* = 46) awake craniotomies.

**Figure 1.**
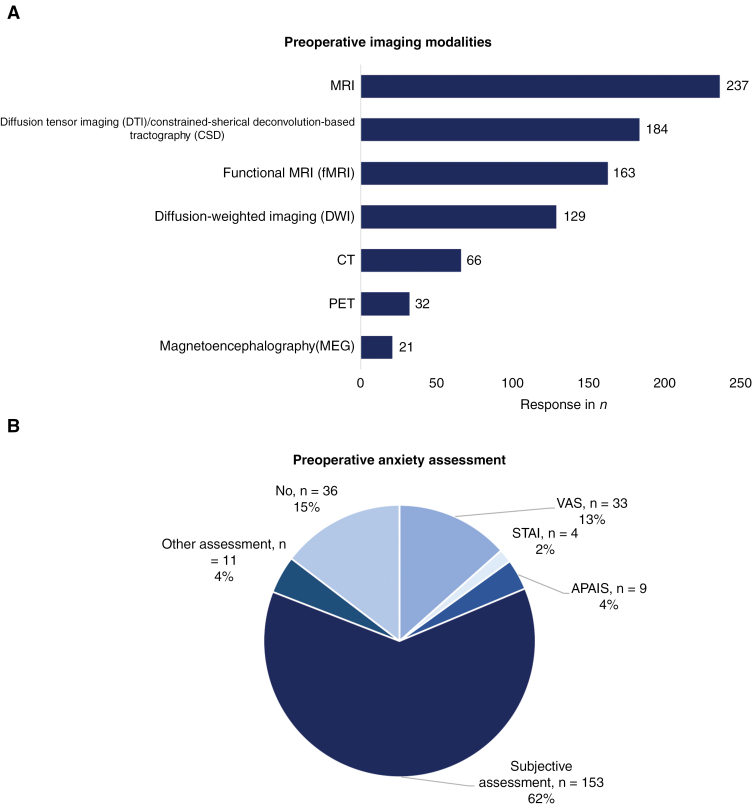
Preparation for awake craniotomy: imaging modalities and preoperative anxiety assessment.

**Figure 2.**
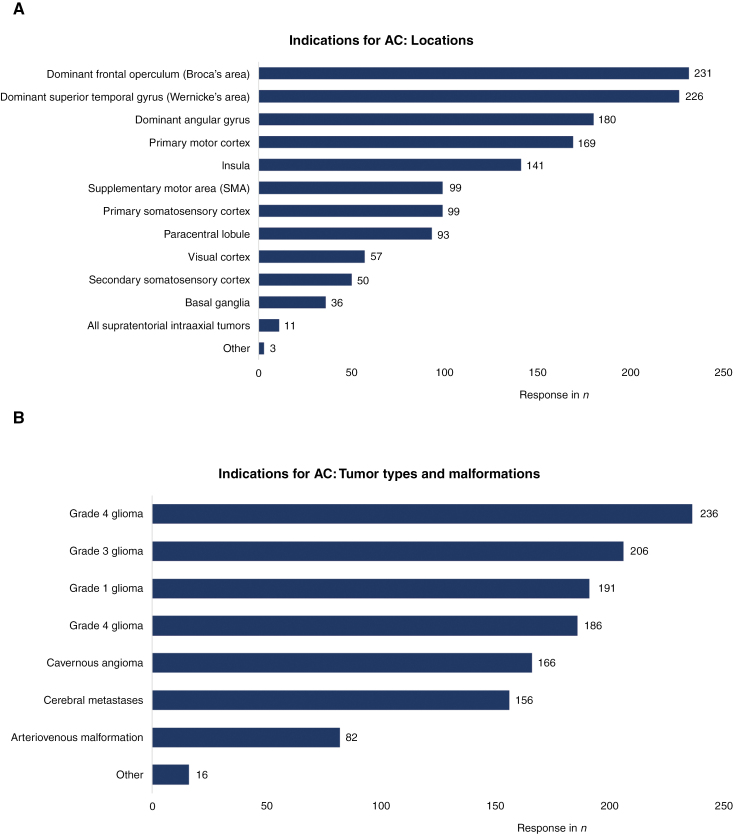
Indications for awake craniotomy, locations, and tumor types or malformations.

In terms of preoperative imaging modalities, significant heterogeneity existed among respondents. MRI and functional MRI (fMRI) were most often used (96.3%, *n* = 237; 66.3%, *n* = 163), while magnetoencephalography (MEG) was least likely to be used in preoperative imaging (8.5%, *n* = 21). Other methods that were used included diffusion-weighted imaging DWI (*n* = 129), diffusion tensor imaging (DTI) or constrained-spherical deconvolution-based tractography (CSD, *n* = 184), and CT (*n* = 66). Preoperative anxiety was assessed by 85.4% of the respondents, with 62.2% through subjective assessment and 18.7% with the use of a local protocol (Figure 1). Tumors that were found eligible for awake craniotomies included grade 1 glioma (77.6%), grade 2 glioma (95.9%), grade 3 glioma (83.7%), grade 4 glioma (75.6%), cerebral metastases (63.4%), and cavernous angioma (67.5%) (Figure 2). Eighty-two respondents (33.3%) selected arteriovenous malformation as suitable for awake surgery.

The main “functional” areas that were most often associated with awake surgery by respondents were the dominant frontal operculum (Broca’s area) (*n* = 231, 93.9%), the dominant superior temporal gyrus (Wernicke’s area) (*n* = 226, 91.9%), the dominant angular gyrus (*n* = 180, 73.2%), and the primary motor cortex (*n* = 169, 68.7%) (Figure 2). Other locations included the insula (*n* = 141, 57.3%), paracentral lobule (*n* = 93, 37.8%), and the primary somatosensory cortex (n = 90, 40.2%). A minority of respondents opted for basal ganglia (*n* = 36, 14.6%) or “all supratentorial intraaxial tumors” (*n* = 11, 4.5%). Overall responses demonstrated high variability (*P* < .0001), as shown in Table 9 (Data Supplementary 2).

**Figure 3.**
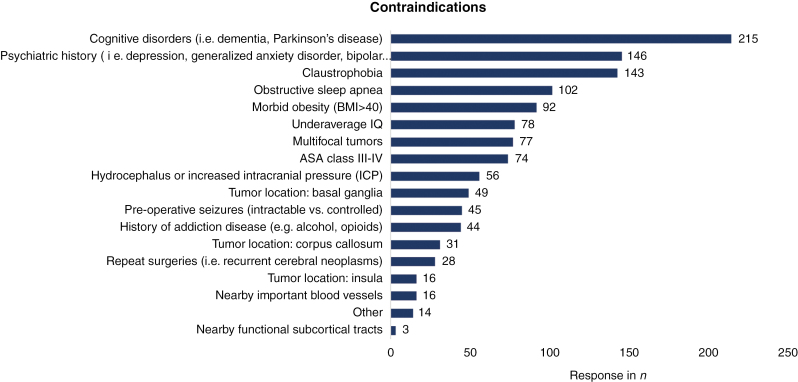
Contraindications for awake surgery.

Respondents varied significantly in their opinions regarding contraindications for awake surgery (*P* < .0001) (Figure 3) (Data Supplementary 2: Table 3). Cognitive disorders (*n* = 215, 88.5%), claustrophobia (*n* = 143, *n* = 58.8%), and psychiatric history (*n* = 146, 60.1%) were most often indicated as contraindications for this technique.

The respondents’ preoperative decision-making was assessed for “general” factors and “patient and psychological” factors (Data Supplementary 2: Table 3). “General” factors were clinical parameters such as the WHO grade of the tumor, comorbidities, and patient functioning, whereas “patient and psychological” factors encompassed psychosocial factors. For “general” factors, respondents attributed the highest importance to “location and eloquence” (median 5 [IQR: 5–5]) and “patient functioning” (median 4 [IQR: 4–5]). Responses differed most on age (mean 2.94, SD = 1.22), patient concerns (median 4 [3–5]), and tumor grade (median 3 [IQR: 1–3.25]). Concerning psychosocial factors, survey responses showed that the patient’s social circumstances had the least weight in the decision to operate awake rather than under general anesthesia (median 2 [IQR: 1–3]). Other psychosocial parameters, such as patient preference and the ability to return to the patient’s current profession had a greater impact on the decision-making process (median 4, [IQR: 3–5]).

Our study also investigated the influence of age in determining whether to perform awake surgery. A total of 45.9% (*n* = 113) of respondents regarded age as an important factor due to the increasing risk of failure to perform reliable intraoperative cognitive assessments associated with increasing age. Another reason, selected by 26.4% of respondents, was a difference in treatment goals for younger and older patients. A total of 28.9% of respondents found age an important factor due to the risk of surgical complications associated with increased age. Finally, a substantial group of neurosurgeons did not consider age an important factor (35.8%). Overall, all differences between perspectives on age were significant (*P* < .0001, Data Supplementary 2: Table 3, question 12) but no significant differences were found in subgroup analyses.

### Balance Between Neurological Function and EOR

A large majority of respondents (*n* = 196, 96.1%) found that “minor cognitive deficits were not acceptable under any circumstances” when asked if such deficits might facilitate gross-total resection (GTR) (Data Supplementary 2, Table 8). Also, respondents were not willing to compromise on function to attain GTR for isolated cognitive domains: executive function (mean 2.99, SD = 1.12), complex attention (mean 2.98, SD = 1.08), social cognition (mean 2.90, SD = 1.03), learning and memory (median 3, IQR = 2–3), language (median 2, IQR = 1–3), and perceptual-motor function (median 2, IQR = 1–3). When asked which factors were important in the decision to compromise on neurocognitive function, the following factors were most influential: career (median 5, IQR = 4–5), family role (median 4, IQR = 4–5), younger age (median 4, IQR = 3–5), and preoperative KPS score 90–100 (median 4, IQR = 3–5). There was relatively a relatively tight range in importance attributed to career and family roles, both with IQR’s of 4–5. Surgeries pertaining to cerebral metastases and preoperative neurological morbidity yielded a high range in attributed importance, with respective standard deviations of 1.25 and 1.14. Questions regarding cessation of the awake procedure showed a similar trend. The patient’s job and/or career (median 4, IQR = 3–5) was most influential in the decision to stop the awake procedure, and, again, re-resection of a tumor (mean 2.91, SD = 1.17) and surgery of a metastasized tumor (mean 3.04, SD = 1.30), showed the highest variability in response. Neurosurgeons also differed vastly in the attribution of importance to preoperative neurological performance in the decision to stop the awake surgery (mean 3.34, SD = 1.22). This was also true for tumor-related factors such as metastasis (mean 3.04, SD = 1.30) and reresection of a tumor (mean 2.91, SD = 1.17). An overview of other factors influencing termination of the awake procedure—such as the patient’s job or career, the patient’s social circumstances, and multifocality—are presented in Data Supplementary 2 (Table 9).

### Neurological Deficits

**Figure 4.**
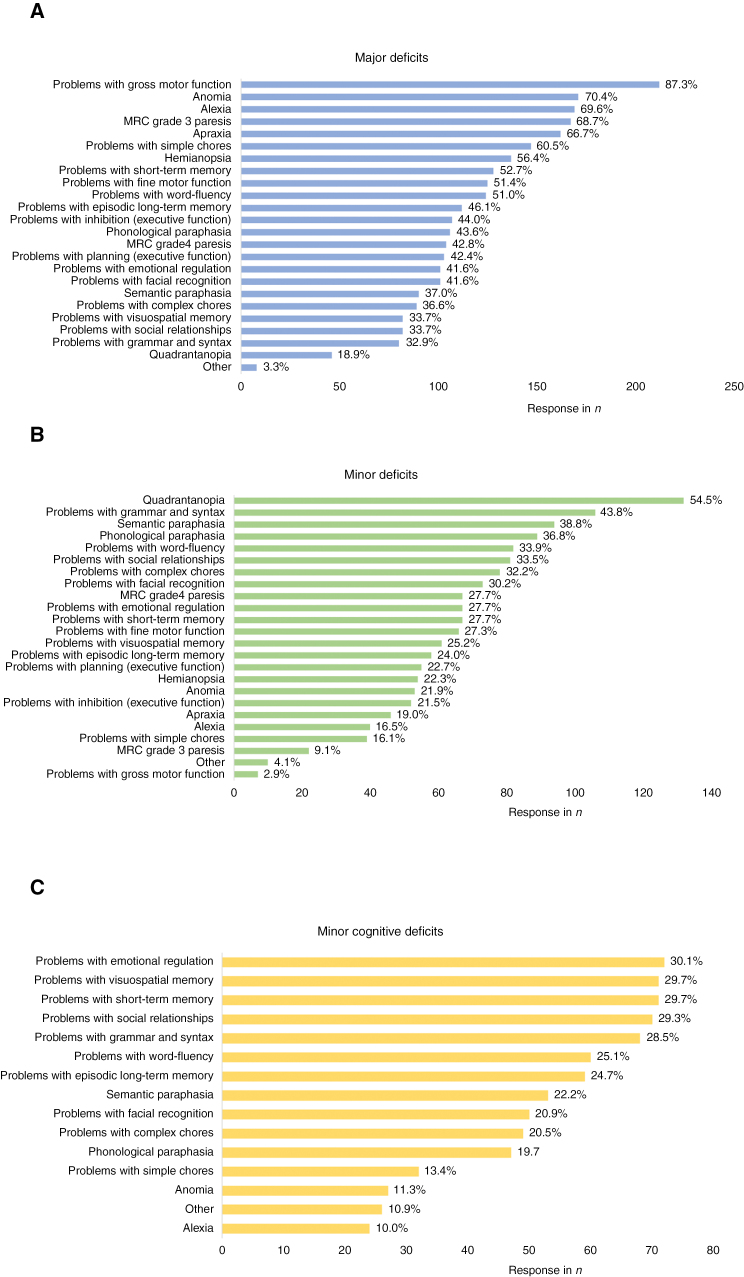
Interpretation of neurological deficits.

**Figure 5.**
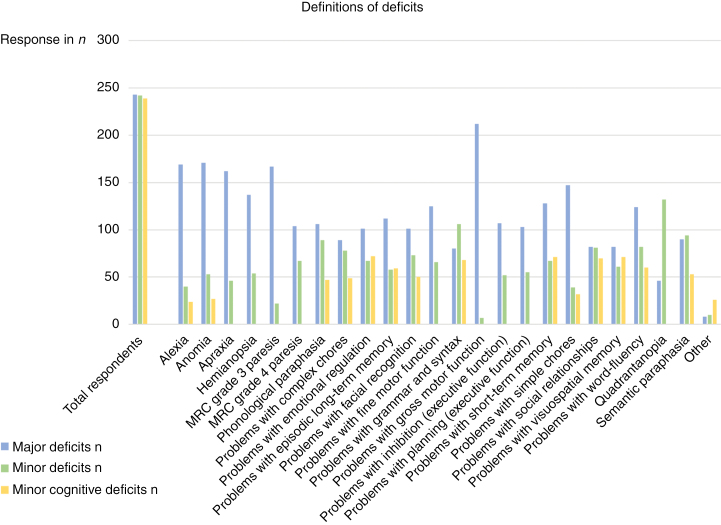
Definitions of all categories: major deficits (Level I), minor deficits (Level II), and minor cognitive deficits.

Most respondents indicated that the maximum extent of expected deficits was discussed with the patient before surgery (*n* = 240, 98.0%) (Data Supplementary 2, Table 3). Overall, the majority of respondents attributed impairments in the language and motor domain to “major” deficits (Figures 4 and 5). On the other hand, the results showed a significant overlap between “major” and “minor cognitive” deficits (Figures 4 and 5). This overlap included problems with emotional regulation, episodic long-term memory, grammar and syntax, social relationships, and visuospatial memory. “Minor” and “minor cognitive” deficits also had considerable overlap in response, mainly regarding alexia, problems with emotional regulation, episodic long-term memory, short-term memory, simple chores, social relationships, visuospatial memory, and word fluency. Examination per category shows that most respondents defined “major” deficits as loss of gross motor function (*n* = 212, 87.2%), anomia (*n* = 171, 70.4%), alexia (*n* = 169, 69.5%), MRC grade 3 paresis (*n* = 167, 68.7%), and apraxia (*n* = 162, 66.7%). Minor deficits included quadrantanopia (*n* = 132, 54.5%), problems with grammar and syntax (*n* = 106, 43.8%), semantic paraphasia (*n* = 94, 38.8%), and phonological paraphasia (*n* = 89, 36.8%), whereas minor cognitive deficits included problems with emotional regulation (*n* = 72, 30.1%), visuospatial memory (*n* = 71, 29.7%), and short-term memory (*n* = 71, 29.7%). An overview per category of deficits can be found in Figures 4 and 5, Data Supplementary 2: Table 4-6 and Table 9.

### Termination of Resection

**Figure 6.**
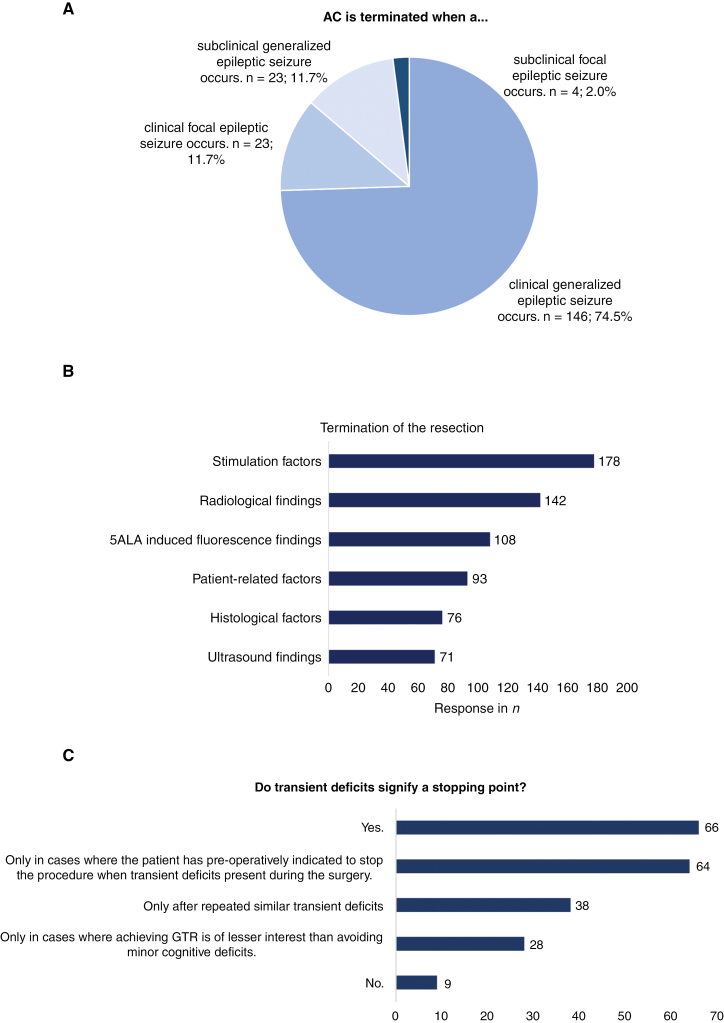
Factors that influence the decision to terminate the resection.

A variety of factors influenced the neurosurgeon’s decision to terminate the resection (Figure 6). Significant differences were found in the way intraoperative seizures affected the decision-making of the neurosurgeon: AC was terminated after a clinical generalized seizure (*n* = 146, 74.5%); after a clinical focal seizure (*n* = 23, 11.7%); after a subclinical generalized seizure (*n* = 23, 11.7%); and, after a subclinical seizure (*n* = 4, 2.0%). Last, responses varied notably regarding transient deficits: 32.1% (*n* = 66) of the neurosurgeons found transient deficits an absolute stopping point and 63.4% (*n* = 130) only stopped the procedure in certain circumstances. Reasons for stopping after transient deficits included situations in which repeated similar transient deficits occur (*n* = 38, 18.5%); when attaining GTR is of lesser interest than avoiding minor cognitive deficits (*n* = 28, 13.7%); or, where the patient has preoperatively indicated to stop the procedure when transient deficits present during the surgery (*n* = 64, 31.2%).

### Quality of Life (QoL) Assessment

QoL was evaluated postoperatively by 82.4% (*n* = 164) of the neurosurgeons in the form of subjective assessment (*n* = 102, 51.3%) or through formal questionnaires: SF36 questionnaire (*n* = 46, 23.1%), EQ-5D (*n* = 24, 12.1%), EORTC QLQ BN20 (*n* = 19, 9.5%), and EORT QLQ C30 (*n* = 17, 8.5%).

### Academic Versus Nonacademic Respondents

To assess the influence of the type of institution, subgroup analyses were made between neurosurgeons from academic and nonacademic institutions. Comparison between academic and nonacademic institutions did not yield notable differences, apart from seizure management and QoL assessment. In total, significant differences were found for 2 of the 72 questions (Data Supplementary 2: Table 8, question 14; and Table 10, question 25). Academic neurosurgeons were less likely to terminate awake surgery after a focal clinical seizure occurs (OR: 0.39 (0.16–0.95), *P* = .0335). They were also more likely to use formal QoL assessments postoperatively, specifically, the EORTC QLQ BN20 (OR: 4.58 (1.02–20.45), *P* = 0.03).

### European Versus North American Respondents

For 7 out of 72 questions, significant differences between European and North American neurosurgeons were found (Data Supplementary 2). European neurosurgeons were more likely to use PET in preparation for awake surgery (OR: 7.25 (2.54–20.70), *P* < .0001). They were less likely to assess anxiety through subjective assessment (OR: 0.19 (0.10–0.36), *P* < .0001). European neurosurgeons reported the secondary somatosensory cortex suitable for AC more often than North American surgeons (OR: 4.97 (2.04–12.12), *P* = .002). They were more likely to select ASA class III-IV and repeat surgeries for recurrent neoplasms as contraindications (OR: 3.14 (1.63–6.08), *P* = .0005; OR: 5.58 (2.07–15.09), *P* = .0002). European respondents less often reported clinical focal and generalized epileptic seizures as indications to stop awake surgery (OR: 0.14 (0.07–0.30), *P* < .0001; OR: 0.02 (0.01–0.06), *P* < .0001). A significant difference was found in postoperative QoL assessment: European neurosurgeons were more likely to use the EORTC QLQ C30 compared to their North American colleagues (OR: 9.20 (1.90–44.30), *P* = .002).

## Discussion

AC is a balancing act between achieving maximal EOR and minimal postoperative deficits. Previous clinical surveys have investigated the decision-making process regarding AC and found significant heterogeneity in the decision to perform awake surgery and the type of approach—defensive or aggressive.^17,22^ These studies have elucidated factors that influence the EOR. Meanwhile, the other arm of the balance—deficit—has currently not been defined by the literature. A previous survey conducted by the ENCRAM Consortium^22^ found that the majority of surveyed neurosurgeons (*n* = 212, 77.5%) (*P* < .0001) reported neurological morbidity as free text in electronic patient systems, but 61.6% of the respondents (*n* = 212) would prefer a standardized neurological assessment scale. Our study aimed to create such a scale by categorizing postoperative deficits according to their suspected severity. The results of the clinical survey reflected the importance of deficits in the decision-making process regarding awake surgery: nearly all neurosurgeons (98.0%) discuss the maximum extent of expected deficits with the patient. Moreover, the majority of them (96.1%) would not strive for GTR if it meant incurring minor cognitive deficits. The most important finding, however, was the significant overlap between “major” and “minor” or “minor cognitive deficits.” Analysis across deficit categories showed that most overlap existed in the domains of executive function, social cognition, and vision. It is likely that this causes considerable variation in perioperative decision-making. Moreover, the respondents’ interpretation of “minor” and “minor cognitive” deficits did not differ significantly from one another. This suggests that these categories may be merged to form a binary system with solely “major” and “minor” deficits, or “level I” and “level II” deficits.

To further understand the AC decision-making process, we also examined its international practice. Results showed significant heterogeneity in several domains, including indications, anxiety assessment, seizure management, and termination of resection. Large variability was seen in the influence of tumor grade in decision-making. This may reflect the history of awake surgery and its gradual international implementation; initially used for epilepsy surgery, AC was later applied to low-grade gliomas and subsequently to high-grade gliomas.^1^ Recent literature has demonstrated that awake surgery could be an effective approach for high-grade gliomas to attain higher EOR, lower residual tumor volume, improved functional outcomes, and improved survival outcomes.^10,11,23–25^ A similar trend can be observed for the surgical management of cerebral metastases, although the concept of deliberately incomplete resection of a cerebral metastasis is not completely validated in current neuro-oncology practice. This survey found that 68.3% of respondents regarded metastases as suitable for awake surgery.^26^ Additionally, the decision to terminate awake surgery in case of metastasized tumors showed high variability among neurosurgeons. A recent systematic review, however, has demonstrated that awake surgery is safe and viable for metastases.^26^ Our results may reflect a slow adaptation of the current literature into practice.

On a different note, this survey investigated the influence of preoperative factors, such as age and patient functioning, with respect to postoperative deficits. Factors that were regarded as most influential were “location and eloquence” (median 5 [IQR: 5–5]) and “patient functioning” (median 4 [IQR: 4–5]), whereas responses varied mostly with regard to age (mean 2.94, SD = 1.22) and tumor grade (median 3 [IQR: 1–3.25]). These findings are in line with a similar survey performed by Gerritsen et al.^17^ in 2022. In the survey, neurosurgeons were asked to offer their surgical strategy—defensive versus aggressive—in four cases with variations in parameters such as age, ASA or KPS score, location and eloquence, preoperative morbidity, and comorbidities. Here, “location and eloquence” (mean = 4.3) and “patient functioning” (mean = 4.2) were deemed more influential than “age” (mean = 3.4). Thus, location and eloquence as well as patient functioning seem to carry more weight in the decision-making process than age.

The results of the survey also show that reliable intraoperative cognitive assessment is crucial in the decision-making process. First, the main contraindications for AC—cognitive disorders and psychiatric history—are conditions that hinder effective intraoperative communication and cognitive assessment. Second, most respondents found age a limiting factor because of the increased risk of failure for intraoperative tests. The importance of reliable intraoperative communication and testing is consistent with the literature. Currently, the main difficulties during awake surgery are communication issues with the patient, and these problems are the main reason for the failure of awake surgery.^27,28^ Over time, intraoperative protocols have improved to overcome these difficulties: Gogos et al.^1^ showed that practical solutions can resolve intraoperative communication issues. Effectively treated psychiatric disorders, for example, need not pose difficulty during surgery.^1^ Results of this survey regarding contraindications may thus be seen as a parameter for implementation of the most recent updates in awake surgery.

Neurosurgeons also differed in practice regarding preoperative anxiety assessment: 18.7% used an established protocol, while 62.2% assessed anxiety subjectively, and European surgeons were more likely to use formal protocols compared to American surgeons. Awake surgery is known to be well-tolerated by patients and patient satisfaction is high across different continents.^29,30^ Simultaneously, the literature is sparse on the effect of undergoing awake surgery on the patient’s psychological well-being and there are certain studies that report negative psychological experiences following AC. Anxiety, for example, seems to play a role in the pain experience. Bala et al^31^ found that preoperative stress is associated with intraoperative stress and pain (Spearon’s rho = 0.722, *P* = .000; Spearon’s rho = 0.368, *P* = .014).^31^ Additionally, general anxiety positively correlated with preoperative stress, intraoperative stress, and pain (Spearon’s rho = 0.470, *P* = .000; Spearon’s rho = 0.617, *P* = .001, Spearon’s rho = 0.600, *P* = .001).^31^ In 2013, Milian et al.^32^ explored the mental health of patients after AC by assessing the frequency and effects of psychological symptoms, such as reliving the surgery or nightmares, on health-related quality of life (HRQOL). Two out of nineteen patients had symptoms that resembled posttraumatic stress disorder (PTSD). The study also found female sex, young age, and experiences of intense anxiety as predisposing factors for negative psychological sequelae after awake surgery. These results suggest two opportunities in research and clinical practice. First of all, anxiety assessment preoperatively may be helpful in selecting patients who will tolerate AC and might be an indicator of intraoperative anxiety. The latter can be helpful in providing medical and psychological guidance throughout the operation, for example, in the form of psychological coaching. Secondly, monitoring mental health multiple times after surgery can give an idea of coping with awake surgery over time, which again may predict the type of guidance and support patients need over time. Overall, it should be considered that, although the literature is sparse on this side subject, awake surgery can be a major life event for patients which comes with a need for medical and psychological help until well after the surgery.

Our study also showed interresponse variability regarding transient deficits. For one-third of the respondents, transient deficits indicate an absolute stopping point (for resection in the corresponding sector of the tumor), while the remaining group found it dependent on circumstances, such as repetition of the deficit. Hamberger et al.^33^ found similar heterogeneity regarding the “operational definition” of language errors during cortical mapping. A systematic review by Collée et al.^34^ investigated the influence of intraoperative speech and language deficits on language outcome and showed a significant relationship between intraoperative anomia (OR = 2.09, *P* = .015) and acute postoperative language deficits. This may suggest that the definition of “transient deficit” may be weighted according to neurological function. For completeness, the interpretation of “transient deficit” should factor in other parameters, such as the time until transient deficit during the surgery, the number of previous transient deficits, tumor location, residual tumor volume, vital parameters, and more. This study was limited by its survey design as former parameters are bound to be influenced by recall bias. Thus, future studies should on the one hand focus on the interpretation of “transient deficit” while including intraoperative factors, while on the other hand categorize different types of transient deficits and study their association with postoperative outcomes.

Along similar lines, our findings on intraoperative seizure management deserve extensive research. It should be taken into account that the results of this survey reflect the clinical approach of neurosurgeons to intraoperative seizures. The finding that European surgeons less often discontinue awake surgery when a seizure occurs compared to their North American counterparts should be interpreted with caution. Due to recall bias, this study was unable to include factors that might be relevant, such as time until seizure, resected tumor volume, and stimulation parameters. Similar to the interpretation of “transient deficits,” this finding may serve as a stepping stone for future research on intraoperative seizure management with a focus on intraoperative factors.

Apart from heterogeneity in AC practice, this survey also elucidated common ground. A common theme was the importance of language and motor skills. Neurosurgeons were not likely to compromise on language and motor function in order to attain GTR. They also attributed most deficits entailing language and motor skills as “major” deficits. Thirdly, the most eligible tumor locations included Broca’s area, Wernicke’s area, and locations involved in motor function. Our findings may reflect the current state of knowledge regarding AC for language and motor function. Literature on AC for these neurological functions is more vast compared to other cognitive domains,^35–37^ such as executive function,^38–40^ and social cognition.^41,42^ This notion is supported by the findings of Rofes et al.^43^: cognitive assessments mainly included language and speech assessments, such as object naming, and found a lesser use of tests for emotional regulation. Another study, by Nakajima et al,^41^ investigated the impact of several cognitive domains on QoL in patients undergoing AC and found that verbal fluency, motor function, and executive function were associated with improved QoL.^41^ Currently, intraoperative cognitive test strategies are investigated to accurately assess executive function, for example by using a modified Stroop test and electrocorticography.^38–40^ Opportunity lies in creating protocols to intraoperatively assess executive function and social cognition in order to attain optimal surgical outcomes and QoL.

### Limitations and Strengths

The main limitation of this study is sampling bias. Dissemination of the online survey via Western networks often leads to a skew of the results towards high-income countries. Our results mainly include North American respondents, with a total of 175 (44.3%) neurosurgeons practicing in the United States. Due to the nature of the distribution, response rates could not be calculated per network. A large part of responses (25.5%) originated from regions outside of Europe and North America that were not specifically targeted via the neurosurgical networks. However, the generalizability of subgroup analyses is limited to European and North American neurosurgeons. Finally, as mentioned before, the interpretation of “transient deficits” and intraoperative seizure management is limited by the study design and warrants further research that includes intraoperative parameters such as stimulation settings, time until transient deficit or seizure, residual volume, et cetera.

Strengths of the study include the scale of distribution, the number of responses, and the depth of the questions. Moreover, this is the first study to evaluate major and minor deficits. The survey demonstrates significant heterogeneity regarding the interpretation of “graded” deficits, which calls for international conversation to define the categories. Lastly, this survey builds onto the model of decision-making with EOR on one side and neurological deficits on the other. Neurosurgeons were asked on which cognitive domains and under which circumstances they may be more willing to compromise on neurocognitive function if a GTR is attained. This offered a unique insight into the decision-making process. Moreover, the online and anonymous character of the survey are advantages as it may have led to honest yet “unpopular” answers on such sensitive statements.

Following this study, future research should be focused on building a decision-making model that balances EOR and postoperative deficits by adding the patients’ perspectives. The opportunity lies in surveying patients on their interpretation of “major” and “minor” deficits. Additionally, patients should be asked if they accept “minor” or “minor cognitive” deficits if this implies a GTR is possible. The patients’ definitions could be compared to the interpretation of neurosurgeons to validate the categories. Other opportunities lie in investigating the association between “minor” and “major” deficits and postoperative QoL. Furthermore, graded deficits might be applied in research that compares supramaximal resection (SMR) outcomes, especially when comparing neurological impairments in patients.^14,15^ A graded deficit system can aid in the comparison of survival after EOR in different IDH1 phenotypes, for example.^18,19^ Last, this study has elucidated discrepancies between neurosurgeons that offer the opportunity to investigate its primary cause. In other words, these differences may be the result of personal opinions or practical constraints. Future studies should consider to investigate potential constraints (ie, equipment, available expertise) to offer a complete picture of decision-making in AC.

## Conclusions

Our results offer insight into the international practice of awake surgery highlight areas that could benefit from international consensus. Substantial heterogeneity was found among neurosurgeons regarding perioperative decision-making, patient selection, anxiety assessment, postoperative QoL assessment, and interpretation of transient deficits. Moreover, neurosurgeons differed in their opinion on the categorization of postoperative deficits. To our knowledge, this survey is the first in its attempt to aid in grading and standardization of neurological deficits using level I and level II deficits. International consensus on a standardized neurological deficit system may aid in assessing the benefit of surgery in neuro-oncology patients. Furthermore, it can be helpful when comparing surgical outcomes across neuro-oncological studies. This is becoming increasingly relevant with the arrival of new surgical techniques (supramaximal resection) and assessment of the potential benefit of surgery in subgroups of patients when weighed against risk of functional loss. 

## Supplementary Material

vdae206_suppl_Supplementary_Data

vdae206_suppl_Supplementary_Materials
