# Supplementary material for: Level I and II deficits—A clinical survey on international practice of awake craniotomy and definitions of postoperative “major” and “minor” deficits
Source: Neurooncol Adv. 2024 Nov 30;6(1):vdae206. doi: 10.1093/noajnl/vdae206 (PMC11647522; doi:10.1093/noajnl/vdae206)
Supplement: vdae206_suppl_Supplementary_Data [file vdae206_suppl_Supplementary_Data.docx]

**Level I and Level II deficits - A clinical survey on international practice of awake craniotomy and definitions of postoperative “major” and “minor” deficits**

**1. Baseline characteristics**

1. Country
2. Region [As defined by WHO region]

- North America (United States, Canada)
- South America (Latin America)
- European region
- Eastern Mediterranean region
- South-East Asia Region
- Western Pacific Region
- African Region

1. Institute

- Academic practice/university hospital
- Non-academic practice/community hospital
- Private hospital
- Other

1. Training level

- Neurosurgeon, >5 years of experience
- Neurosurgeon, <5 years of experience

1. Average number of awake craniotomies performed per year

- 0
- 1-5
- 6-20
- 21-50
- 51-100
- 100+

**PART 1: Pre-operative**

1. What pre-operative imaging modalities are used to assess the relationship between functional tissue and tumor?

(*Please select all options that apply.*)

- MRI
- Magnetoencephalography (MEG)
- Diffusion-weighted imaging (DWI)
- Diffuse tensor imaging (DTI)/constrained-spherical deconvolution-based tractography (CSD)
- Functional MRI (fMRI)
- CT
- PET

1. Is the preoperative anxiety of the patient assessed?

- Yes:
- Visual analog scale (VAS)
- State-Trait Anxiety Inventory (STAI)
- Amsterdam Preoperative Anxiety and Information Scale (APAIS)
- Subjective assessment of anxiety
- Other, please specify: …
- No

1. In your opinion, what tumor types and malformations are eligible for awake craniotomy? [According to: World Health Organization (WHO) Brain Tumor Grades]

*(Please select all options that apply.*)

- Grade 1 glioma,
- Grade 2 glioma
- Grade 3 glioma
- Grade 4 glioma
- Cerebral metastases
- Cavernous angioma
- Arteriovenous malformation
- Other, please specify: …

1. In your opinion, which eloquent areas are indications for awake craniotomy?

(*Please select all options that apply.*)

- Primary somatosensory cortex
- Secondary somatosensory cortex
- Primary motor cortex
- Paracentral lobule
- Supplementary motor area (SMA)
- Dominant frontal operculum (Broca’s area)
- Dominant superior temporal gyrus (Wernicke’s area)
- Dominant angular gyrus
- Insula
- Basal ganglia
- Visual cortex
- All supratentorial intraaxial tumors
- Other, please specify: …

1. Which factors influence your decision to perform an awake craniotomy over craniotomy under general anesthesia?

(*Please indicate the influence on a Likert scale of 1-5, for each factor independently, where 5 indicates the strongest impact on your decision.*)

- Location and eloquence
- Patient functioning (e.g. KPS or ECOG)
- Comorbidities (e.g. ASA)
- Preoperative neurological morbidity
- Preoperative tumor size
- Age
- Patient concerns (claustrophobia, anxiety, etc)
- WHO-grade of the tumor

1. How does the patient’s age play a role in your decision making?

(*Please select all options that apply.*)

- Increasing risk of surgical complications with increasing age.
- Increasing risk of neurological complications with increasing age.
- Increasing risk of inability to perform reliable intra-operative cognitive assessments.
- The treatment’s goals differ for younger and older GBM patients.
- Older patients tend to prefer one of the surgical modalities.
- Age is not a factor in the decision to perform an awake craniotomy.
- Other, please specify: …

1. Which psychological and social patient factors favor awake craniotomy over craniotomy under general anesthesia?

(*Please indicate the influence on a Likert scale of 1-5, for each factor independently, with 5 being the strongest indicator to perform awake craniotomy.*)

- Patient’s preference
- Patient’s social circumstances
- Patient’s ability to return to current profession

1. What are contraindications for awake craniotomy?

(*Please select all options that apply.*)

- Cognitive disorders (i.e. dementia, Parkinson’s disease)
- Claustrophobia
- Psychiatric history (i.e. depression, generalized anxiety disorder, bipolar disorder)
- History of addiction disease (e.g. alcohol, opioids)
- Pre-operative seizures (intractable vs. controlled)
- Morbid obesity (BMI>40)
- Obstructive sleep apnea
- Multifocal tumors
- Nearby important blood vessels
- Nearby functional subcortical tracts
- Tumor location: insula
- Tumor location: basal ganglia
- Tumor location: corpus callosum
- Hydrocephalus or increased intracranial pressure (ICP)
- Underaverage IQ
- ASA class III-IV
- Repeat surgeries (i.e. recurrent cerebral neoplasms)
- Other, please specify: …

1. Is the maximum extent of expected deficits discussed with the patient before surgery?

- Yes
- No
- Other, please specify: …

1. What is your definition of ‘major’ or ‘Level I’ deficits?

(*Please select all options that apply.*)

- Anomia, i.e. word-finding impairment
- Apraxia, i.e. impairment of motor aspect of speech
- Alexia, i.e. inability to comprehend written material
- Phonological paraphasia, i.e. substitution of a word with a nonword or incorrect word while half of the original word is preserved
- Semantic paraphasia, i.e. substitution of a word with a similar word that resembles the original meaning
- Problems with word-fluency
- Problems with grammar and syntax
- Problems with simple chores, i.e. bathing
- Problems with complex chores, i.e. cooking
- Problems with social relationships, i.e. recognizing emotions in other people
- Problems with facial recognition
- Problems with short-term memory, i.e. recalling a phone number
- Problems with episodic long-term memory, i.e. recalling life events
- Problems with emotional regulation, i.e. uncontrolled emotional outbursts
- Problems with visuospatial memory, i.e. recalling shapes, colors, movement and location of an object
- Problems with inhibition (executive function)
- Problems with planning (executive function)
- Hemianopsia
- Quadrantanopia
- Problems with fine motor function, i.e. holding a pencil
- Problems with gross motor function, i.e. walking
- MRC grade 3 paresis, i.e. able to actively move against gravity
- MRC grade 4 paresis, i.e. able to actively move against gravity and some resistance
- Other, please specify: …

1. What is your definition of ‘minor’ or ‘Level II’ deficits?

(*Please select all options that apply.*)

- Anomia, i.e. word-finding impairment
- Apraxia, i.e. impairment of motor aspect of speech
- Alexia, i.e. inability to comprehend written material
- Phonological paraphasia, i.e. substitution of a word with a nonword or incorrect word while half of the original word is preserved
- Semantic paraphasia, i.e. substitution of a word with a similar word that resembles the original meaning
- Problems with word-fluency
- Problems with grammar and syntax
- Problems with simple chores, i.e. bathing
- Problems with complex chores, i.e. cooking
- Problems with social relationships, i.e. recognizing emotions in other people
- Problems with facial recognition
- Problems with short-term memory, i.e. recalling a phone number
- Problems with episodic long-term memory, i.e. recalling life events
- Problems with emotional regulation, i.e. uncontrolled emotional outbursts
- Problems with visuospatial memory, i.e. recalling shapes, colors, movement and location of an object
- Problems with inhibition (executive function)
- Problems with planning (executive function)
- Hemianopsia
- Quadrantanopia
- Problems with fine motor function, i.e. holding a pencil
- Problems with gross motor function, i.e. walking
- MRC grade 3 paresis, i.e. able to actively move against gravity
- MRC grade 4 paresis, i.e. able to actively move against gravity and some resistance
- Other, please specify: …

1. What is your definition of ‘minor cognitive deficits’?

(*Please select all options that apply.*)

- Anomia, i.e. word-finding impairment
- Alexia, i.e. inability to comprehend written material
- Phonological paraphasia, i.e. substitution of a word with a nonword or incorrect word while half of the original word is preserved
- Semantic paraphasia, i.e. substitution of a word with a similar word that resembles the original meaning
- Problems with word-fluency
- Problems with grammar and syntax
- Problems with simple chores, i.e. bathing
- Problems with complex chores, i.e. cooking
- Problems with social relationships, i.e. recognizing emotions in other people
- Problems with facial recognition
- Problems with short-term memory, i.e. recalling a phone number
- Problems with episodic long-term memory, i.e. recalling life events
- Problems with emotional regulation, i.e. uncontrolled emotional outbursts
- Problems with visuospatial memory, i.e. recalling shapes, colors, movement and location of an object
- Other, please specify: …

**PART 2: Intra-operative**

1. Could in certain circumstances minor post-operative cognitive deficits be acceptable, if GTR is thereby attained?

- Yes
- No, under no circumstances are minor cognitive deficits acceptable.

1. For which cognitive domain might you be more willing to compromise on its function if a gross-total resection (GTR) is attained?

(*Please indicate the influence on a Likert scale of 1-5, for each factor independently, with 5 indicating maximum ability to compromise on function.*)

- Executive function, i.e. planning of a day trip
- Complex attention, i.e. sustained attention to a task
- Social cognition, i.e. understanding social interactions
- Learning and memory, i.e. being able to recall and reproduce a story
- Language, i.e. speech and word-finding
- Perceptual-motor function, i.e. walking and throwing a ball

1. Under which circumstance might you be less willing to compromise on neurocognitive function if a gross-total resection (GTR) is attained?

(*Please indicate the influence on a Likert scale of 1-5, for each circumstance independently. where 5 would indicate absolute avoidance of minor deficits.*)

- The patient has a strong wish to return to former job, in which neurocognitive function plays a significant role.
- The patient has a substantial family role.
- The patient has poor social support/network.
- The procedure is a re-resection of the tumor.
- In case of resection of a cerebral metastasis.
- In case of an older patient.
- In case of a younger patient.
- Pre-operative KPS 90-100.
- Pre-operative KPS <80.
- No pre-operative neurological morbidity
- Significant pre-operative neurological morbidity

1. Based on which factors do you decide to terminate the resection?

(*Please select all options that apply.*)

- Radiological findings (e.g. maximum resection based on neuronavigation with/without DTI)
- Ultrasound findings
- 5ALA induced fluorescence findings
- Histological factors (e.g. macroscopic maximum resection)
- Stimulation factors (e.g. deficits)
- Patient-related factors (e.g. fatigue)

1. What patient and tumor factors influence your decision to stop the awake procedure?

(*Please indicate the influence on a Likert scale of 1-5, for each factor independently, with 5 maximally favoring a stop.*)

- Age
- Pre-operative neurological performance
- Pre-operative KPS
- Pre-operative ASA
- Patient’s job/career
- Patient’s social circumstances (i.g. family role)
- Multifocality
- Metastasis
- Re-resection
- Other, please specify: …

1. Do transient deficits signify a stopping point?

*(Please select the statement that you most strongly agree with.)*

- Yes.
- Yes, but only after repeated similar transient deficits.
- Yes, but only in cases where the patient has pre-operatively indicated to stop the procedure when transient deficits present during the surgery.
- Yes, but only in cases where achieving GTR is of lesser interest than avoiding minor cognitive deficits.
- No.

1. In the case of epileptic seizures, when is the awake craniotomy terminated?

- AC is terminated when a clinical generalized epileptic seizure occurs.
- AC is terminated when a clinical focal epileptic seizure occurs.
- AC is terminated when a subclinical generalized epileptic seizure occurs.
- AC is terminated when a subclinical focal epileptic seizure occurs.

**PART 3: Postoperative**

1. Is there an outpatient quality of life assessment performed after surgery?

(*Please select all options that apply.)*

- EORTC QLQ BN20
- EORTC QLQ C30
- EQ-5D
- SF36 questionnaire
- Other formal quality of life assessment, please specify: …
- Subjective quality of life assessment
- No assessment is performed.
