# Supplementary material for: Level I and II deficits—A clinical survey on international practice of awake craniotomy and definitions of postoperative “major” and “minor” deficits
Source: Neurooncol Adv. 2024 Nov 30;6(1):vdae206. doi: 10.1093/noajnl/vdae206 (PMC11647522; doi:10.1093/noajnl/vdae206)
Supplement: vdae206_suppl_Supplementary_Materials [file vdae206_suppl_Supplementary_Materials.docx]

**Level I and Level II deficits - A clinical survey on international practice of awake craniotomy and definitions of postoperative “major” and “minor” deficits**

M. Vooijs, Dr. F. C. Robertson, S. E. Blitz, Prof. dr. P. Schucht, Dr. C. Jungk, MD, Prof. dr. S.M. Krieg, Prof. dr. S. De Vleeschouwer, Prof. dr. A.J.P.E. Vincent, Dr. M. S. Berger, Dr. B.V. Nahed, Prof. dr. M. L. B. Broekman, Dr. J. K. W. Gerritsen

Table 1. Baseline characteristics

Table 2. Respondents per country

Table 3. Preoperative phase

Table 4. Definitions of ‘major’ or ‘Level I’ deficits

Table 5. Definitions of ‘minor’ or ‘Level II’ deficits

Table 6. Definitions of ‘minor cognitive deficits’

Table 7. Neurological deficits – summary

Table 8. Factors influencing termination of the surgery

Table 9. Intraoperative decision-making: factors influencing gross-total resection and stop of procedure

Table 10. Postoperative quality of life assessment

**Legend**

**Bold** Statistically significant after Bonferroni correction

* *Fisher’s exact test*

**Table 1. Baseline characteristics**

| **Characteristics** | **Number of responses (n)**  **(n_total_=395)** | **(%)** |
| --- | --- | --- |
| **Region (World Health Organization)** |  |  |
| North America | 191 | 50.1 |
| South America | 27 | 7.1 |
| European Region | 93 | 24.4 |
| Eastern Mediterranean Region | 14 | 3.7 |
| South East Asia Region | 45 | 11.8 |
| Western Pacific Region | 6 | 1.6 |
| African Region | 5 | 1.3 |
| **Institute** |  |  |
| Academic hospital | 247 | 64.8 |
| Non-academic hospital | 70 | 18.4 |
| Private hospital | 55 | 14.4 |
| Other | 9 | 2.4 |
| **Training** |  |  |
| >5 years | 335 | 87.9 |
| <5 years | 46 | 12.1 |
| **Number of awake craniotomies performed** |  |  |
| 0 | 23 | 6.0 |
| 1-5 | 168 | 44.1 |
| 6-20 | 131 | 34.4 |
| 21-50 | 46 | 12.1 |
| 50-100 | 10 | 2.6 |
| 100+ | 3 | 0.8 |

**Table 2. Respondents per country**

| **Characteristics** | **Number of responses (n)**  **(n_total_ = 395)** | **(%)** |
| --- | --- | --- |
| Unknown | 15 | 3.8 |
| Argentina | 3 | 0.7 |
| Austra | 1 | 0.2 |
| Australia | 13 | 3.2 |
| Austria | 3 | 0.7 |
| Belgium | 12 | 3.0 |
| Brazil | 8 | 2.0 |
| Canada | 17 | 4.3 |
| Chile | 4 | 1.0 |
| China | 3 | 0.7 |
| Colombia | 5 | 1.2 |
| Czech.Replubic | 2 | 0.5 |
| Denmark | 2 | 0.5 |
| Ecuador | 1 | 0.2 |
| Finland | 2 | 0.5 |
| France | 4 | 1.0 |
| Germany | 5 | 1.2 |
| Greece | 3 | 0.7 |
| India | 18 | 4.5 |
| Indonesia | 1 | 0.2 |
| Iran | 1 | 0.2 |
| Israel | 4 | 1.0 |
| Italy | 13 | 3.2 |
| Malaysia | 2 | 0.5 |
| Mexico | 6 | 1.5 |
| Morocco | 2 | 0.5 |
| Netherlands | 13 | 3.2 |
| Nepal | 1 | 0.2 |
| Nigeria | 2 | 0.5 |
| Norway | 1 | 0.2 |
| Pakistan | 4 | 1.0 |
| Philippines | 3 | 0.7 |
| Poland | 1 | 0.2 |
| Portugal | 4 | 1.0 |
| South Korea | 2 | 0.5 |
| Romania | 1 | 0.2 |
| Saudi Arabia | 5 | 1.2 |
| Serbia | 1 | 0.2 |
| Spain | 8 | 2.0 |
| Sudan | 1 | 0.2 |
| Switzerland | 3 | 0.7 |
| Taiwan | 1 | 0.2 |
| Thailand | 1 | 0.2 |
| Turkey | 10 | 2.5 |
| United Arab Emirates | 2 | 0.5 |
| United Kingdom | 6 | 1.5 |
| United States of America | 175 | 44.3 |

**Table 3. Preoperative phase**

Q6. What preoperative imaging modalities are used to assess the relationship between functional tissue and tumor?

*Multiple options.*

n = 246

| **Answer options** | **Overall response, n (%)** | ***P* value** | **Odds ratio for academic vs non-academic, private, and other (95% CI)** | ***P* value** | **Odds ratio for European vs North American surgeons (95% CI)** | ***P* value** |
| --- | --- | --- | --- | --- | --- | --- |
| • MRI • Magnetoencephalography (MEG) • Diffusion-weighted imaging (DWI) • Diffusion tensor imaging (DTI)/constrained-spherical deconvolution-based tractography (CSD) • Functional MRI (fMRI) • CT • PET | 237 (96.3)  21 (8.5)  129 (52.4)  184 (74.8)  163 (66.3)  66 (26.8)  32 (13.0) | _a/b_ **<.0001**  _a/c_ **<.0001**  _a/d_ **<.0001**  _a/e_ **<.0001**  _a/f_ **<.0001**  _a/g_ **<.0001**  _b/c_ **<.0001**  _b/d_ **<.0001**  _b/e_ **<.0001**  _b/f_  <**.0001**  _b/g_ 0.1096  _c/d_ **<.0001**  _c/e_ 0.0018  _c/f_ **<.0001**  _d/e_ 0.0378  _d/f_ **<.0001**  _d/g_ **<.0001**  _e/f_ **<.0001**  _e/g_ **<.0001**  _f/g_ **0.0001** | 0.57 (0.11-2.81)  5.14 (1.16-22.63)  0.83 (0.49-1.42)  1.40 (1.77-2.56)  1.06 (0.60-1.85)  0.89 (0.49-1.61)  1.09 (0.49-2.43) | 0.3840*  0.0171  0.4930  0.2617  0.8415  0.6985  0.8230 | 0.58 (0.11-2.97)  0.83 (0.27-2.54)  1.76 (0.97-3.22)  0.97 (0.49-1.94)  0.67 (0.36-1.25)  0.84 (0.41-1.70)  7.25 (2.54-20.70) | 0.3977  0.7401  0.0636  0.9203  0.2088  0.6242  **<.0001** |

Q7. Is the preoperative anxiety of the patient assessed?

*Multiple options.*

n = 246

| **Answer options** | **Overall response, n (%)** | ***P* value** | **Odds ratio for academic vs non-academic, private, and other (95% CI)** | ***P* value** | **Odds ratio for European vs North American surgeons (95% CI)** | ***P* value** |
| --- | --- | --- | --- | --- | --- | --- |
| • Yes:   - Visual analog scale (VAS) - State-Trait Anxiety Inventory (STAI) - Amsterdam Preoperative Anxiety and Information Scale (APAIS) - Subjective assessment of anxiety - Other, please specify: …   • No | 33 (13.4)  4 (1.6)  9 (3.7)  153 (62.2)  11 (4.5)  36 (14.6) | _a/b_ **<.0001**  _a/c_ **0.0001**  _a/d_ **<.0001**  _a/f_ 0.6985  _b/c_ 0.1594  _b/d_ **<.0001**  _b/f_ **<.0001**  _c/d_ **<.0001**  _c/f_ **<.0001**  _d/f_ **<.0001** | 0.98 (0.45-2.13)  NA  1.75 (0.36-8.62)  0.81 (0.47-1.41)  2.28 (0.48-10.80)  0.85 (0.40-1.78) | 1.00  0.3840*  0.4624  0.2370  0.6629 | 3.03 (1.23-7.43)  0.40 (0.10-1.63)  NA  NA  0.19 (0.10-0.36)  2.32 (0.50-10.70)  1.84 (0.80-4.24) | 0.0123  0.1896  **<.0001**  0.2345*  0.1473 |

Q8. In your opinion, what tumor types and malformations are eligible for awake craniotomy?

*Multiple options.*

n = 246

| **Answer options** | **Overall response, n (%)** | ***P* value** | **Odds ratio for academic vs non-academic, private, and other (95% CI)** | ***P* value** | **Odds ratio for European vs North American surgeons (95% CI)** | ***P* value** |
| --- | --- | --- | --- | --- | --- | --- |
| • Grade 1 glioma,  • Grade 2 glioma  • Grade 3 glioma  • Grade 4 glioma  • Cerebral metastases  • Cavernous angioma  • Arteriovenous malformation  • Other, please specify: … | 191 (77.6)  236 (95.9)  206 (83.7)  186 (75.6)  156 (63.4)  166 (67.5)  82 (33.3)  16 (6.5) | _a/b_ **<.0001**  _a/c_ 0.0864  _a/d_ 0.5967  _a/e_ 0.0005  _a/f_ 0.0115  _a/g_ **<.0001**  _b/c_ **<.0001**  _b/d_ **<.0001**  _b/e_ **<.0001**  _b/f_  **<.0001**  _b/g_ **<.0001**  _c/d_ 0.0251  _c/e_ **<.0001**  _c/f_ **<.0001**  _d/e_ 0.0033  _d/f_ 0.0457  _d/g_ **<.0001**  _e/f_ 0.3428  _e/g_ **<.0001**  _f/g_ **<.0001** | 2.01 (1.09-3.72)  2.11 (0.59-7.49)  1.45 (0.72-2.90)  1.13 (0.61-2.09)  0.80 (0.46-1.42)  0.82 (0.46-1.46)  0.78 (0.45-1.37)  NA | 0.0249  0.2005*  0.2987  0.6985  0.4583  0.4976  0.3865 | 0.93 (0.46-1.86)  0.27 (0.07-1.15)  0.45 (0.20-1.02)  0.47 (0.24-0.93)  1.10 (0.59-2.04)  1.01 (0.54-1.89)  0.72 (0.38-1.34)  NA | 0.8230  0.0668*  0.0525  0.0273  0.7641  1.00  0.2965 |

Q9. In your opinion, which eloquent areas are indications for awake craniotomy?

*Multiple options.*

n = 246

| **Answer options** | **Overall response, n (%)** | ***P* value** | **Odds ratio for academic vs non-academic, private, and other (95% CI)** | ***P* value** | **Odds ratio for European vs North American surgeons (95% CI)** | ***P* value** |
| --- | --- | --- | --- | --- | --- | --- |
| • Primary somatosensory cortex  • Secondary somatosensory cortex  • Primary motor cortex  • Paracentral lobule  • Supplementary motor area (SMA)  • Dominant frontal operculum (Broca’s area)  • Dominant superior temporal gyrus (Wernicke’s area)  • Dominant angular gyrus  • Insula  • Basal ganglia  • Visual cortex  • All supratentorial intraaxial tumors  • Other, please specify: … | 99 (40.2)  50 (20.3)  169 (68.7)  93 (37.8)  99 (40.2)  231 (93.9)  226 (91.9)  180 (73.2)  141 (57.3)  36 (14.6)  57 (23.2)  11 (4.5)  3 (1.2) | _a/b_ **<.0001**  _a/c_ **<.0001**  _a/d_ 0.5777  _a/e_ 1.00  _a/f_ **<.0001**  _a/g_ **<.0001**  _a/h_ **<.0001**  _a/i_ 0.0002  _a/j_ **<.0001**  _a/k_ **<.0001**  _a/l_ **<.0001**  _b/c_ **<.0001**  _b/d_ **<.0001**  _b/e_ **<.0001**  _b/f_  **<.0001**  _b/g_ **<.0001**  _b/h_ **<.0001**  _b/i_ **<.0001**  _b/j_ 0.0966  _b/k_ 0.4424  _b/l_ **<.0001**  _c/d_ **<.0001**  _c/e_ **<.0001**  _c/f_ **<.0001**  _c/g_ **<.0001**  _c/h_ 0.2753  _c/i_ 0.0089  _c/j_ **<.0001**  _c/k_ **<.0001**  _c/l_ **<.0001**  _d/e_ 0.5777  _d/f_ **<.0001**  _d/g_ **<.0001**  _d/h_ **<.0001**  _d/i_ **<.0001**  _d/j_ **<.0001**  _d/k_ 0.0004  _d/l j_ **<.0001**  _e/f_ **<.0001**  _e/g_ **<.0001**  _e/h_ **<.0001**  _e/i_ 0.0002  _e/j_ **<.0001**  _e/k_ **<.0001**  _e/l_ **<.0001**  _f/g_ 0.3802  _f/h_ **<.0001**  _f/i_ **<.0001**  _f/j_ **<.0001**  _f/k_ **<.0001**  _f/l_ **<.0001**  _g/h_**<.0001**  _g/i_ **<.0001**  _g/j_ **<.0001**  _g/k_ **<.0001**  _g/l_ **<.0001**  _h/i_ 0.0002  _h/j_ **<.0001**  _h/k_ **<.0001**  _h/l_ **<.0001**  _i/j_ **<.0001**  _i/k_ **<.0001**  _i/l_ **<.0001**  _j/k_ 0.0156  _j/l_ 0.0001  _k/l_ **<.0001** | 0.59 (0.32-1.08)  0.68 (0.33-1.39)  0.35 (0.16-0.72)  1.22 (0.69-2.18)  0.73 (0.40-1.34)  0.66 (0.13-3.40)  0.66 (0.17-2.53)  1.81 (0.94-3.51)  1.24 (0.68-2.27)  2.14 (0.77-6.00)  0.66 (0.33-1.33)  0.98 (0.24-4.07)  NA | 0.0838  0.2921  0.0034  0.4884  0.3125  0.4746*  0.3980*  0.0736  0.4839  0.1389  0.2453  0.6186* | 2.79 (1.39-5.59)  4.97 (2.04-12.12)  1.39 (0.67-2.88)  2.18 (1.09-4.33)  3.42 (1.70-6.90)  0.82 (0.13-5.08)  0.53 (0.10-2.76)  1.28 (0.55-2.95)  1.27 (0.63-2.57)  1.10 (0.38-3.21)  2.29 (0.98-5.34)  3.84 (0.68-21.69)  NA | 0.0033  **0.0002**  0.3771  0.0253  0.0005  0.5852*  0.3629  0.5657  0.4976  0.5334  0.0509  0.1190* |

Q10. Which factors influence your decision to perform an awake craniotomy over craniotomy under general anesthesia?

*Likert scale, where 1= least impact, 5 = strongest impact.*

n = 246

| **Answer options** | **Overall response, median [IQR]** | ***P* value** |
| --- | --- | --- |
| • Location and eloquence  • Patient functioning (e.g. KPS or ECOG)  • Comorbidities (e.g. ASA)  • Preoperative neurological morbidity  • Preoperative tumor size  • Age  • Patient concerns (claustrophobia, anxiety, etc.)  • WHO-grade of the tumor | Median 5 [5-5]  Median 4 [4-5]  Mean 3.19 (SD=1.17)  Mean 3.79 (SD=1.03)  Mean 2.76 (SD=1.18)  Mean 2.94 (SD=1.22)  Median 4 [3-5]  Median 3 [1-3.25] | NA |

Q11. Which psychological and social patient factors favor awake craniotomy over craniotomy under general anesthesia?

*Likert scale, where 1= least impact, 5 = strongest impact.*

n = 245

| **Answer options** | **Overall response, median [IQR]** | ***P* value** |
| --- | --- | --- |
| • Patient’s preference  • Patient’s social circumstances  • Patient’s ability to return to current profession | Median 4 [3-5]  Median 2 [1-3]  Median 4 [3-5] | NA |

Q12. How does the patient’s age play a role in your decision making?

*Multiple options.*

n = 246

| **Answer options** | **Overall response, n (%)** | ***P* value** | **Odds ratio for academic vs non-academic, private, and other (95% CI)** | ***P* value** | **Odds ratio for European vs North American surgeons (95% CI)** | ***P* value** |
| --- | --- | --- | --- | --- | --- | --- |
| • Increasing risk of surgical complications with increasing age.  • Increasing risk of neurological complications with increasing age.  • Increasing risk of inability to perform reliable intra-operative cognitive assessments.  • The treatment’s goals differ for younger and older GBM patients.  • Older patients tend to prefer one of the surgical modalities.  • Age is not a factor in the decision to perform an awake craniotomy.  • Other, please specify: … | 71 (28.9)  39 (15.9)  113 (45.9)  65 (26.4)  9 (3.7)  88 (35.8)  8 (3.3) | _a/b_ **0.0005**  _a/c_ **<.0001**  _a/d_ 0.5430  _a/e_ **<.0001**  _a/f_ 0.1009  _b/c_ **<0.001**  _b/d_ **0.0040**  _b/e_ **<0.001**  _b/f_ **<0.001**  _c/d_ **<0.001**  _c/e_ **<0.001**  _c/f_ 0.0218  _d/e_ **<0.001**  _d/f_  **0.0250**  _e/f_ **<0.001** | 1.36 (0.75-2.49)  0.75 (0.37-1.52)  0.88 (0.51-1.49)  1.04 (0.57-1.90)  0.98 (0.24-4.03)  1.08 (0.62-1.89)  NA | 0.3125  0.4237  0.6242  0.8875  0.6160*  0.7773 | 0.90 (0.48-1.71)  0.96 (0.44-2.09)  1.26 (0.69-2.28)  0.93 (0.47-1.82)  NA  1.21 (0.66-2.22)  NA | 0.7518  0.9203  0.4463  0.8415  0.5430 |

Q13. What are contraindications for awake craniotomy?

*Multiple options.*

n = 243

| **Answer options** | **Overall response, n (%)** | ***P* value** | **Odds ratio for academic vs non-academic, private, and other (95% CI)** | ***P* value** | **Odds ratio for European vs North American surgeons (95% CI)** | ***P* value** |
| --- | --- | --- | --- | --- | --- | --- |
| • Cognitive disorders (i.e. dementia, Parkinson’s disease)  • Claustrophobia  • Psychiatric history (i.e. depression, generalized anxiety disorder, bipolar disorder)  • History of addiction disease (e.g. alcohol, opioids)  • Pre-operative seizures (intractable vs. controlled)  • Morbid obesity (BMI>40)  • Obstructive sleep apnea  • Multifocal tumors  • Nearby important blood vessels  • Nearby functional subcortical tracts  • Tumor location: insula  • Tumor location: basal ganglia  • Tumor location: corpus callosum  • Hydrocephalus or increased intracranial pressure (ICP)  • Underaverage IQ  • ASA class III-IV  • Repeat surgeries (i.e. recurrent cerebral neoplasms)  • Other, please specify: … | 215 (88.5)  143 (58.8)  146 (60.1)  44 (18.1)  45 (18.5)  92 (37.9)  102 (42.0)  77 (31.7)  16 (6.6)  3 (1.2)  16 (6.6)  49 (20.2)  31 (12.8)  56 (23.0)  78 (32.1)  74 (30.5)  28 (11.5)  14 (5.8) | _a/b_ **<.0001**  _a/c_ **<.0001**  _a/d_ **<.0001**  _b/c_ 0.7773  _b/d_ **<.0001**  _b/e_ **<.0001**  _c/d_ **<.0001**  _c/f_ **<.0001**  _d/f_ **<.0001** | 0.64 (0.26-1.60)  0.63 (0.36-1.10)  0.86 (0.50-1.49)  1.21 (0.59-2.47)  0.48 (0.25-0.94)  1.54 (0.87-2.71)  0.90 (0.52-1.54)  0.80 (0.45-1.41)  0.81 (0.28-2.30)  0.98 (0.09-10.99)  1.51 (0.47-4.84)  0.65 (0.34-1.24)  0.55 (0.26-1.18)  0.85 (0.45-1.59)  0.97 (0.55-1.72)  0.67 (0.38-1.19)  0.62 (0.28-1.38)  NA | 0.3428  0.1003  0.5902  0.5967  0.0298  0.1371  0.6985  0.2629  0.6892  0.7000*  0.4839  0.1265  0.1206  0.6101  0.9203  0.1692  0.2351 | 0.80 (0.34-1.92)  0.70 (0.39-1.29)  1.84 (1.00-3.41)  1.89 (0.86-4.15)  2.22 (1.03-4.80)  0.92 (0.49-1.71)  0.99 (0.54-1.83)  0.86 (0.44-1.68)  2.21 (0.57-8.51)  0.85 (0.08-9.50)  2.21 (0.57-8.51)  0.86 (0.40-1.86)  1.29 (0.56-2.99)  1.13 (0.55-2.36)  1.74 (0.90-3.38)  3.14 (1.63-6.08)  5.58 (2.07-15.09)  NA | 0.6171  0.2560  0.0497  0.1102  0.0390  0.7913  1.00  0.6629  0.2037*  0.6902  0.2037*  0.6985  0.5541  0.7290  0.0985  **0.0005**  **0.0002** |

Q14. Is the maximum extent of expected deficits discussed with the patient before surgery?

n = 245

| **Answer options** | **Overall response, n (%)** | **Odds ratio for academic vs non-academic, private, and other (95% CI)** | ***P* value** | **Odds ratio for European vs North American surgeons (95% CI)** | ***P* value** |  | ***P* value** |
| --- | --- | --- | --- | --- | --- | --- | --- |
| • Yes  • No  • Other, please specify: … | 240 (98.0)  5 (2.0)  0 | NA |  | NA |  |  |  |

**Table 4. Definitions of ‘major’ or ‘Level I’ deficits**

Q15. What is your definition of ‘major’ or ‘Level I’ deficits?

*Multiple options.*

n = 243

| **Answer options** | **Overall response, n (%)** |
| --- | --- |
| • Anomia, i.e. word-finding impairment  • Apraxia, i.e. impairment of motor aspect of speech  • Alexia, i.e. inability to comprehend written material  • Phonological paraphasia, i.e. substitution of a word with a nonword or incorrect word while half of the original word is preserved  • Semantic paraphasia, i.e. substitution of a word with a similar word that resembles the original meaning  • Problems with word-fluency  • Problems with grammar and syntax  • Problems with simple chores, i.e. bathing  • Problems with complex chores, i.e. cooking  **•** Problems with social relationships, i.e. recognizing emotions in other people  • Problems with facial recognition  • Problems with short-term memory, i.e. recalling a phone number  • Problems with episodic long-term memory, i.e. recalling life events  • Problems with emotional regulation, i.e. uncontrolled emotional outbursts  • Problems with visuospatial memory, i.e. recalling shapes, colors, movement and location of an object  • Problems with inhibition (executive function)  • Problems with planning (executive function)  • Hemianopsia  • Quadrantanopia  • Problems with fine motor function, i.e. holding a pencil  • Problems with gross motor function, i.e. walking  • MRC grade 3 paresis, i.e. able to actively move against gravity  • MRC grade 4 paresis, i.e. able to actively move against gravity and some resistance  • Other, please specify: … | 171 (70.4)  162 (66.7)  169 (69.5)  106 (43.6)  90 (37.0)  124 (51.0)  80 (32.9)  147 (60.5)  89 (36.6)  82 (33.7)  101 (41.6)  128 (52.7)  112 (46.1)  101 (41.6)  82 (33.7)  107 (44.0)  103 (42.4)  137 (56.4)  46 (18.9)  125 (51.4)  212 (87.2)  167 (68.7)  104 (42.8)  8 (3.3) |

**Table 5. Definitions of ‘minor’ or ‘Level II’ deficits**

Q16. What is your definition of ‘minor’ or ‘Level II’ deficits?

*Multiple options.*

n = 242

| **Answer options** | **Overall response, n (%)** |
| --- | --- |
| • Anomia, i.e. word-finding impairment  • Apraxia, i.e. impairment of motor aspect of speech  • Alexia, i.e. inability to comprehend written material  • Phonological paraphasia, i.e. substitution of a word with a nonword or incorrect word while half of the original word is preserved  • Semantic paraphasia, i.e. substitution of a word with a similar word that resembles the original meaning  • Problems with word-fluency  • Problems with grammar and syntax  • Problems with simple chores, i.e. bathing  • Problems with complex chores, i.e. cooking  **•** Problems with social relationships, i.e. recognizing emotions in other people  • Problems with facial recognition  • Problems with short-term memory, i.e. recalling a phone number  • Problems with episodic long-term memory, i.e. recalling life events  • Problems with emotional regulation, i.e. uncontrolled emotional outbursts  • Problems with visuospatial memory, i.e. recalling shapes, colors, movement and location of an object  • Problems with inhibition (executive function)  • Problems with planning (executive function)  • Hemianopsia  • Quadrantanopia  • Problems with fine motor function, i.e. holding a pencil  • Problems with gross motor function, i.e. walking  • MRC grade 3 paresis, i.e. able to actively move against gravity  • MRC grade 4 paresis, i.e. able to actively move against gravity and some resistance  • Other, please specify: … | 53 (21.9)  46 (19.0)  40 (16.5)  89 (36.8)  94 (38.8)  82 (33.9)  106 (43.8)  39 (16.1)  78 (32.2)  81 (33.5)  73 (30.2)  67 (27.7)  58 (24.0)  67 (27.7)  61 (25.2)  52 (21.5)  55 (22.7)  54 (22.3)  132 (54.5)  66 (27.3)  7 (2.9)  22 (9.1)  67 (27.7)  10 (4.1) |

**Table 6. Definitions of ‘minor cognitive deficits’**

Q17. What is your definition of ‘minor cognitive deficits’?

*Multiple options.*

n = 239

| **Answer options** | **Overall response, n (%)** |
| --- | --- |
| • Anomia, i.e. word-finding impairment  • Alexia, i.e. inability to comprehend written material  • Phonological paraphasia, i.e. substitution of a word with a nonword or incorrect word while half of the original word is preserved  • Semantic paraphasia, i.e. substitution of a word with a similar word that resembles the original meaning  • Problems with word-fluency  • Problems with grammar and syntax  • Problems with simple chores, i.e. bathing  • Problems with complex chores, i.e. cooking  • Problems with social relationships, i.e. recognizing emotions in other people  • Problems with facial recognition  • Problems with short-term memory, i.e. recalling a phone number  • Problems with episodic long-term memory, i.e. recalling life events  • Problems with emotional regulation, i.e. uncontrolled emotional outbursts  • Problems with visuospatial memory, i.e. recalling shapes, colors, movement and location of an object  • Other, please specify: … | 27 (11.3)  24 (10.0)  47 (19.7)  53 (22.2)  60 (25.1)  68 (28.5)  32 (13.4)  49 (20.5)  70 (29.3)  50 (20.9)  71 (29.7)  59 (24.7)  72 (30.1)  71 (29.7)  26 (10.9) |

**Table 7. Neurological deficits – summary**

| **Neurological deficits** | **Δ Major - Minor %** | **χ2** | **p-value** | **Δ Major - Minor cog. %** | **χ2** | **p-value** | **Δ Minor - Minor cog. %** | **χ2** | **p-value** |
| --- | --- | --- | --- | --- | --- | --- | --- | --- | --- |
| Alexia | 53.02 | 138.98 | **<.0001** | 59.51 | 177.71 | **<.0001** | 6.49 | 4.39 | 0.0362 |
| Anomia | 48.47 | 114.61 | **<.0001** | 59.07 | 173.72 | **<.0001** | 10.60 | 9.75 | **0.0018** |
| Apraxia | 47.66 | 112.43 | **<.0001** | 66.67 |  |  | 19.01 |  |  |
| Hemianopsia | 34.06 | 58.94 | **<.0001** | 56.38 |  |  | 22.31 |  |  |
| MRC grade 3 paresis | 59.63 | 181.3 | **<.0001** | 68.72 |  |  | 9.09 |  |  |
| MRC grade 4 paresis | 15.11 | 12.13 | **0.0005** | 42.80 |  |  | 27.69 |  |  |
| Phonological paraphasia | 6.84 | 2.36 | 0.1245 | 23.96 | 31.92 | **<.0001** | 17.11 | 17.36 | **<.0001** |
| Problems with complex chores | 4.39 | 1.04 | 0.3078 | 16.12 | 15.33 | **<.0001** | 11.73 | 8.51 | 0.0035 |
| Problems with emotional regulation | 13.88 | 10.31 | **0.0013** | 11.44 | 6.85 | 0.0089 | -2.44 | 0.35 | 0.5541 |
| Problems with episodic long-term memory | 22.12 | 26.07 | **<.0001** | 21.40 | 8.07 | 0.0045 | -0.72 | 0.03 | 0.8625 |
| Problems with facial recognition | 11.40 | 6.85 | **0.0089** | 20.64 | 23.87 | **<.0001** | 9.24 | 5.4 | **0.0201** |
| Problems with fine motor function | 24.17 | 29.67 | **<.0001** | 51.44 |  |  | 27.27 |  |  |
| Problems with grammar and syntax | -10.88 | 6.07 | 0.0138 | 4.47 | 1.13 | 0.2878 | 15.35 | 12.27 | **0.0005** |
| Problems with gross motor function | 84.35 | 348.35 | **<.0001** | 87.24 |  |  | 2.89 |  |  |
| Problems with inhibition (executive function) | 22.55 | 27.97 | **<.0001** | 44.03 |  |  | 21.49 |  |  |
| Problems with planning (executive function) | 19.66 | 21.34 | **<.0001** | 42.39 |  |  | 22.73 |  |  |
| Problems with short-term memory | 24.99 | 31.49 | **<.0001** | 22.97 | 26.22 | **<.0001** | -2.02 | 0.24 | 0.6242 |
| Problems with simple chores | 44.38 | 101 | **<.0001** | 47.10 | 114.52 | **<.0001** | 2.73 | 0.71 | 0.3994 |
| Problems with social relationships | 0.27 | 0 | 1 | 4.46 | 1.11 | 0.2921 | 4.18 | 0.98 | 0.3222 |
| Problems with visuospatial memory | 8.54 | 4.25 | 0.0393 | 4.04 | 0.91 | 0.3401 | -4.50 | 1.22 | 0.2694 |
| Problems with word-fluency | 17.14 | 14.59 | **0.0001** | 25.92 | 34.31 | **<.0001** | 8.78 | 4.45 | 0.0349 |
| Quadrantanopia | -35.62 | 66.2 | **<.0001** | 18.93 |  |  | 54.55 |  |  |
| Semantic paraphasia | -1.81 | 0.17 | 0.6801 | 14.86 | 12.75 | **0.0004** | 16.67 | 15.74 | **<.0001** |
| Other | -0.84 | NA | NA | -7.59 | NA | NA | -6.75 | NA | NA |

**Table 8. Factors influencing termination of the surgery**

Q18. Could in certain circumstances minor post-operative cognitive deficits be acceptable, if GTR is thereby attained?

*n = 204*

| **Answer options** | **Overall response, n (%)** | ***P* value** | **Odds ratio for academic vs non-academic, private, and other (95% CI)** | ***P* value** | **Odds ratio for European vs North American surgeons (95% CI)** | ***P* value** |
| --- | --- | --- | --- | --- | --- | --- |
| • Yes  • No, under no circumstances are minor cognitive deficits acceptable. | 8 (3.9)  196 (96.1) | _a/b_ **<.0001** | NA |  | NA |  |

Q21. Based on which factors do you decide to terminate the resection?

*Multiple options.*

*n = 205*

| **Answer options** | **Overall response, n (%)** | ***P* value** | **Odds ratio for academic vs non-academic, private, and other (95% CI)** | ***P* value** | **Odds ratio for European vs North American surgeons (95% CI)** | ***P* value** |
| --- | --- | --- | --- | --- | --- | --- |
| • Radiological findings (e.g. maximum resection based on neuronavigation with/without DTI)  • Ultrasound findings  • 5ALA induced fluorescence findings  • Histological factors (e.g. macroscopic maximum resection)  • Stimulation factors (e.g. deficits)  • Patient-related factors (e.g. fatigue) | 142 (69.3)  71 (34.6)  108 (52.7)  76 (37.1)  178 (86.8)  93 (45.4) | _a/b_ **<.0001**  _a/c_ 0.0006  _a/d_ **<.0001**  _a/e_ **<.0001**  _a/f_ **<.0001**  _b/c_ 0.0002  _b/d_ 0.0603  _b/e_ **<.0001**  _b/f_ 0.0265  _c/d_ 0.0015  _c/f_ 0.1380  _d/e_ **<.0001**  _d/f_ 0.0880  _e/f_ **<.0001** | 0.44 (0.22-0.88)  1.48 (0.78-2.79)  0.98 (0.54-1.76)  0.72 (0.39-1.31)  1.06 (0.45-2.51)  0.69 (0.39-1.25) | 0.0183  0.2253  1.00  0.2753  0.8875  0.2222 | 0.46 (0.23-0.94)  1.63 (0.84-3.17)  2.00 (1.01-3.96)  1.07 (0.55-2.09)  1.16 (0.41-3.28)  1.03 (0.53-1.97) | 0.0312  0.1502  0.0449  0.8415  0.7773  0.9203 |

Q23. Do transient deficits signify a stopping point?

*n = 205*

| **Answer options** | **Overall response, n (%)** | ***P* value** | **Odds ratio for academic vs non-academic, private, and other (95% CI)** | ***P* value** | **Odds ratio for European vs North American surgeons (95% CI)** | ***P* value** |
| --- | --- | --- | --- | --- | --- | --- |
| • Yes.  • Yes, but only after repeated similar transient deficits.  • Yes, but only in cases where the patient has pre-operatively indicated to stop the procedure when transient deficits present during the surgery.  • Yes, but only in cases where achieving GTR is of lesser interest than avoiding minor cognitive deficits.  • No. | 66 (32.2)  38 (18.5)  64 (31.2)  28 (13.7)  9 (4.4) | _a/b_ 0.0015  _a/c_ 0.8231  _a/d_ **<.0001**  _a/e_ **<.0001**  _b/c_ 0.0030  _b/d_ 0.1785  _b/e_ **<.0001**  _c/d_ **<.0001**  _d/e_ 0.0011 | 1.11 (0.28-4.45)  1.21 (0.66-2.20)  2.17 (0.78-6.04)  0.71 (0.37-1.38)  0.65 (0.30-1.40) | 0.5910*  0.5322  0.1302  0.3149  0.2674 | 0.28 (0.03- 2.38)  1.79 (0.92-3.48)  0.93 (0.35-2.50)  0.83 (0.40-1.73)  0.62 (0.23-1.68) | 0.2061*  0.0828  0.8875  0.6242  0.3428 |

Q24. In the case of epileptic seizures, when is the awake craniotomy terminated?

*n = 196*

| **Answer options** | **Overall response, n (%)** | ***P* value** | **Odds ratio for academic vs non-academic, private, and other (95% CI)** | ***P* value** | **Odds ratio for European vs North American surgeons (95% CI)** | ***P* value** |
| --- | --- | --- | --- | --- | --- | --- |
| • AC is terminated when a clinical generalized epileptic seizure occurs.  • AC is terminated when a clinical focal epileptic seizure occurs.  • AC is terminated when a subclinical generalized epileptic seizure occurs.  • AC is terminated when a subclinical focal epileptic seizure occurs. | 146 (74.5)  23 (11.7)  23 (11.7)  4 (2.0) | _a/b_ **<.0001**  _a/c_ **<.0001**  _a/d_ **<.0001**  _b/c_ 1.00  _b/d_ **0.0001**  _c/d_ **0.0001** | 1.55 (0.79-3.01)  0.39 (0.16-0.95)  1.43 (0.53-3.82)  0.47 (0.07-3.47) | 0.1990  **0.0335**  0.4751  0.3952* | 0.14 (0.07-0.30)  0.02 (0.01-0.06)  0.64 (0.22-1.88)  NA | **<.0001**  **<.0001**  0.4131 |

**Table 9. Intraoperative decision-making: factors influencing gross-total resection and stop of procedure**

| **Question** | **Answer options** | **Overall response, median [IQR]** | ***P* value** |
| --- | --- | --- | --- |
| For which cognitive domain might you be more willing to compromise on its function if a gross-total resection (GTR) is attained?  *Likert scale, where 5 indicates maximum willingness to compromise on function.*  n = 196 | • Executive function, i.e. planning of a day trip  • Complex attention, i.e. sustained attention to a task  • Social cognition, i.e. understanding social interactions  • Learning and memory, i.e. being able to recall and reproduce a story  • Language, i.e. speech and word-finding  • Perceptual-motor function, i.e. walking and throwing a ball | Mean 2.99 (SD=1.12)  Mean 2.98 (SD=1.08)  Mean 2.90 (SD=1.03)  Median 3 [2-3]  Median 2 [1-3]  Median 2 [1-3] | NA |
| Under which circumstance might you be less willing to compromise on neurocognitive function if a gross-total resection (GTR) is attained?  *Likert scale, where 5 indicates absolute avoidance of minor deficits.*  n = 196 | • The patient has a strong wish to return to former job, in which neurocognitive function plays a significant role.  • The patient has a substantial family role.  • The patient has poor social support/network.  • The procedure is a re-resection of the tumor.  • In case of resection of a cerebral metastasis.  • In case of an older patient.  • In case of a younger patient.  • Pre-operative KPS 90-100.  • Pre-operative KPS <80.  • No pre-operative neurological morbidity  • Significant pre-operative neurological morbidity | Median 5 [4-5]  Median 4 [4-5]  Mean 3.64 (SD=1.09)  Mean 2.92 (SD=1.09)  Mean 3.06 (SD=1.25)  Mean 3.21 (SD=1.09)  Median 4 [3-5]  Median 4 [3-5]  Mean 3.22 (SD=1.03)  Median 4 [3-5]  Mean 2.90 (SD=1.14) | NA |
| What patient and tumor factors influence your decision to stop the awake procedure?  *Likert scale, with 5 maximally favoring a stop.*  n = 196 | • Age  • Pre-operative neurological performance  • Pre-operative KPS  • Pre-operative ASA  • Patient’s job/career  • Patient’s social circumstances (i.g. family role)  • Multifocality  • Metastasis  • Re-resection  • Other, please specify: … | Median 2 [1-3]  Mean 3.34 (SD=1.22)  Mean 3.34 (SD=1.11)  Mean 2.78 (SD=1.15)  Median 4 [3-5]  Mean 3.517 (SD=1.10)  Median 4 [3-5]  Mean 3.04 (SD=1.30)  Mean 2.91 (SD=1.17)  38 (15.4) | NA |

**Table 10. Postoperative quality of life assessment**

Q25. Is there an outpatient quality of life assessment performed after surgery?

*Multiple options.*

n = 199

| **Answer options** | **Overall response, n (%)** | ***P* value** | **Odds ratio for academic vs non-academic, private, and other (95% CI)** | ***P* value** | **Odds ratio for European vs North American surgeons (95% CI)** | ***P* value** |
| --- | --- | --- | --- | --- | --- | --- |
| • EORTC QLQ BN20  • EORTC QLQ C30  • EQ-5D  • SF36 questionnaire  • Other formal quality of life assessment, please specify: …  • Subjective quality of life assessment  • No assessment is performed. | 19 (9.5)  17 (8.5)  24 (12.1)  46 (23.1)  10 (5.0)  102 (51.3)  35 (17.6) | _a/b_ 0.7290  _a/c_ 0.4201  _a/d_ 0.0003  _a/e_ 0.0828  _a/f_ <**.0001**  _a/g_ 0.0192  _b/c_ 0.2488  _b/d_ **<.0001**  _b/e_ 0.1626  _b/f_  **<.0001**  _b/g_ 0.0074  _c/d_ 0.0037  _c/e_ 0.0121  _c/f_ **<.0001**  _d/e_ **<.0001**  _d/f_ **<.0001**  _d/g_ 0.1703  _e/f_ **<.0001**  _e/g_ **<.0001**  _f/g_ **<.0001** | 4.58 (1.02-20.45)  3.97 (0.88-17.91)  1.98 (0.71-5.57)  0.51 (0.24-1.07)  4.61 (0.57-37.18)  0.94 (0.52-1.70)  0.51 (0.24-1.07) | **0.0305**  0.0547  0.1871  0.0717  0.1060*  0.8415  0.0697 | 3.35 (0.94-12.02)  9.20 (1.90-44.30)  2.49 (0.87-7.11)  1.64 (0.78-3.42)  NA  0.68 (0.35-1.33)  0.79 (0.33-1.90) | 0.0548  **0.0020**  0.0812  0.1884  0.2617  0.6033 |
